# Secretory IgA as Biomarker for Gastrointestinal Nematodes Natural Infection in Different Breed Sheep

**DOI:** 10.3390/ani13132189

**Published:** 2023-07-04

**Authors:** Verónica Castilla Gómez de Agüero, Elora Valderas-García, Laura González del Palacio, F. Javier Giráldez, Rafael Balaña-Fouce, María Martínez-Valladares

**Affiliations:** 1 Instituto de Ganadería de Montaña, CSIC-Universidad de León, 24346 Grulleros, Spain; vcastg@unileon.es (V.C.G.d.A.); evalg@unileon.es (E.V.-G.); lgonzp@unileon.es (L.G.d.P.); j.giraldez@eae.csic.es (F.J.G.); 2 Departamento de Sanidad Animal, Facultad de Veterinaria, Universidad de León, Campus de Vegazana, 24007 León, Spain; 3 Departamento de Ciencias Biomédicas, Facultad de Veterinaria, Universidad de León, Campus de Vegazana, 24007 León, Spain; rbalf@unileon.es

**Keywords:** GIN, natural infections, IgA, biomarker, nasal secretions, protein disulfide isomerase, L_3_ somatic antigen

## Abstract

**Simple Summary:**

Gastrointestinal nematode (GIN) infections are serious parasitosis that cause disease in grazing livestock. The impact of these parasites is associated with important economic losses related to decreased production and the cost of anthelmintic treatments. Previous studies have reported that GIN infections, mainly those caused by *Teladorsagia circumcincta*, are associated with specific IgA levels. The goal of this study was to characterize IgA levels in naturally infected sheep belonging to Assaf, Castellana, and Churra breeds in different samples (blood, nasal secretions, and saliva). The association between IgA and fecal egg count, breed, and age was also studied. The infection risk according to age and/or breed was measured by a multilevel random intercept model. As a result, the model predicted that breed was not a factor influencing the risk of infection, while age was determinant. On the other hand, this study concludes that nasal secretions could be a useful sample to detect natural infections in young animals from any of the breeds included in this study. Further studies in sheep belonging to other breeds would be interesting in the future to verify this test.

**Abstract:**

Specific IgA antibody has been shown to play an important role in resistance to gastrointestinal nematode (GIN) infections in sheep, particularly in *Teladorsagia circumcincta* parasitosis. In some breeds, negative associations have been shown between IgA levels and worm burden in experimentally infected sheep. In the present study, we have studied the relationship between IgA levels in naturally infected sheep (582 ewes in total; 193 younger than one year old and 389 older than one year old) and fecal egg count (FEC) in the Assaf, Castellana, and Churra breeds. ELISA assays were performed to measure IgA levels against the somatic antigen of *T. circumcincta third larval stage* (L_3_) and a 203-amino-acid fragment of the protein disulfide isomerase from the same GIN species. A multilevel random intercept model was developed to predict the infection risk according to age or breed. Spearman’s correlation rank was used for statistical analysis. The prediction model showed that breed was not an influential factor in this study, although the Assaf breed could be considered slightly more susceptible than the others. In addition, age affected the infection risk, with the young ewes more susceptible to infection than the adult groups, except for the Castellana breed, whose risk of infection was similar at all ages. The most significant positive association was found between FEC and IgA measured in the nasal secretions of young ewes using both antigens (Rho = 0.5; *p* = 0.00); the correlation of FEC with IgA in serum was moderately significant (Rho = 0.306; *p* = 0.00). Comparing both antigens, the protein disulfide isomerase antigen was less reactive than the somatic antigen from L_3_. In conclusion, under natural conditions, specific IgA against GIN was positively associated with FEC in sheep, with nasal secretions from young animals being the sample where this association is stronger, which, therefore, could be used as a marker of infection in further studies.

## 1. Introduction

In temperate countries, the high prevalence of gastrointestinal nematodes (GIN) in small ruminants is a major constraint in sheep production systems, reducing milk, meat, and wool production on farms and affecting their economic performance [1,2,3]. According to the most recent published reports, *Teladorsagia circumcincta* is the most prevalent GIN species in sheep in these regions, followed by *Trichostrongylus* spp., *Haemonchus contortus*, *Oesophagostomum* spp., and *Chabertia ovina* [4,5,6,7]. Broad-spectrum anthelmintic drugs have been used for more than 40 years to effectively control infections. However, their improper administration has led to the appearance and evolution of anthelmintic resistance (AR) [8,9,10]. Owing to the progression of AR worldwide, it has been necessary to develop alternative control methods, such as selective treatment, grazing management, or biological control, among others, to reduce the use of anthelmintic drugs [7,11,12,13,14]. One of the most promising alternative methods is the selection of hosts with a phenotype resistant to GIN infections because it allows for long-lasting and regular control of nematodosis [15]. In the absence of reliable biological markers to recognize resistant or susceptible sheep populations, fecal egg count (FEC) has been the most used method for the identification of hosts with a resistant phenotype. In fact, Martinez-Valladares et al. [16] found a positive and significant correlation between FEC and the number of *T. circumcincta* adult worms in the abomasum (Rho = 0.502; *p* < 0.05) or the number of eggs present in the uterus of adult females (Rho = 0.438; *p* < 0.05). Breeding selection programs based on FEC and its heritability have even been developed in Merino sheep in Australia [17,18]. However, FEC is limited by several factors, including the variation in the fecundity of each GIN species, the sheep breed, the composition of feed, stress, or the host’s immune system condition [19,20]. Moreover, some authors have reported that to obtain reliable FEC results, it is necessary for sheep to have not been treated with AH for at least 14 weeks before the analysis [20,21,22]. Due to these disadvantages, numerous studies have suggested IgA activity as a more convenient marker of infection than FEC [16,23,24]. An increase of IgA activity in abomasal mucosa, plasma, and saliva has been associated with lower FEC in naturally infected Scottish Blackface and Lleyn lambs against the somatic antigen of *T. circumcincta* third (L_3_)- and fourth-stage larvae (L_4_), indicating probable control of the fecundity of adult females by IgA [25,26,27]. This association has also been found in naturally infected Pelibuey lambs when IgA ismeasured against the somatic antigen of *H. contortus* [28]. Under experimental conditions, IgA levels have been measured in serum, saliva, and nasal secretions in adult Churra ewes against the somatic antigen of *T. circumcincta* L_4_, establishing negative associations between FEC and IgA levels [16].

Secretory IgA antibody (IgA) is the most abundant humoral component on the mucosa surface and has been detected in certain biological fluids such as saliva, nasal secretions, and lachrymal glands [29,30]. Helminth infections are known to generate an immunological response where the IgA antibody is secreted, acting asan important mediator to control the infections. IgA is involved in immune elimination and immune exclusion of some pathogens, GINs among them [16,29]. During GIN infections, IgA detects specific antigens and binds to them as part of immune protection, but little is still known about its role [29,31]. Accordingly, several studies are focused on identification of antigens to be used to detect anti-GIN IgA levels [32,33]. The somatic antigen is composed of a great variety of antigenic molecules, protein disulfide isomerase (PDI) among them. This protein has been detected in all larval stages of *T. circumcincta*, *T. colubriformis*, and *H. contortus* [34,35]. PDI is an essential enzyme found in the endoplasmic reticulum whose main role is catalytic, via disulfide bond formation during protein folding, and is overexpressed in the secretory cells; thus, PDI could go through the endoplasmic membrane and be detected in the cell environment [29,36,37]. In a previous study, members of our group identified, isolated, cloned, and tested one of the most conserved antigenic fragments of *T. circumcincta* PDI protein (PDI-Tc) [35]. This specific fragment has a 203 amino acid size and corresponds to one of the activity sites. Subsequently, it was tested to measure specific IgA levels in blood, saliva, and nasal secretions in Churra sheep during a *T. circumcincta* experimental infection [38].

Spain has the highest number of sheep in the European Union, as it isan essential economic sector in rural regions [39]. According to the Spanish Ministry of Agriculture, Fisheries and Food, in the sampled area, Assaf, Castellana, and Churra are the most important sheep breeds in production terms. Castellana and Churra are autochthonous breeds, with the Assaf breed being introduced to improve milk yields. The management system of flocks is mainly semi-extensive, due to the large natural land extension available in the area [40]. The most recent studies showed 100% GIN prevalence, with *T. circumcincta* the most common species (present in 100% of the flocks), followed by *Trichostrongylus* spp. (present in 92% of the flocks) [7].

In order to evaluate the specific IgA response against natural GIN infections in different breeds of sheep—Assaf, Castellana, and Churra—saliva, nasal secretions, and blood were analyzed in young and adult sheep belonging to different flocks from the northwest of Spain. IgA levels were then related to the infection level measured by the FEC. A multilevel random intercept model was developed to explain the variability in the infection. Specific IgA against NGI was positively associated with FEC in sheep; the nasal secretions of young animals provided the samples where this association was stronger, and such samples could therefore be used as a marker of infection. Moreover, the model showed that breed is not an influencing factor in this case, although the Castellana breed is likely more infected than the others. As expected, young animals showed a higher probability of suffering from these infections than adult ewes, with the exception of the Castellana breed, for which all animals showed the same probability regardless ofage.

## 2. Materials and Methods

### 2.1. Animal Selection

The study was carried out on195 ewes younger than oneyearold and 394 adult ewes, for a total of 589. The ewes belonged to the Assaf, Castellana, and Churra breeds and came from 15 commercial flocks situated in the northwest of Spain. All flocks were under semi-intensive management, grazing for at least 6 h per day. The selected animals were not given any deworming treatment for at least three months before sampling and were not pregnant. The Assaf breed is raised for milk production, but the Churra and Castellana breeds are destined for a mixed production of milk and meat. In this area, communal pastures are the most common system, where several flocks share the same grazing lands.

The number of animals, as well as the breed and type of pasture foreach flock, are summarized in Table 1. 

### 2.2. Animal Sampling

Feces were collected directly from the rectum to determine the FEC. The number of eggs per gram of feces (epg) was analyzed using the McMaster technique, with a lower limit of detection of 15 epg [41]. One coproculture per flock was performed for the morphological identification of larval species.

Blood samples were taken from the jugular vein and deposited into glass tubes without anticoagulant (Vacutainer; Barcelona, Spain) to obtain serum. Serum samples were stored frozen (−20 °C) until further analysis [16]. 

Nasal secretions were collected by introducing two cotton swabs (Deltalab; Barcelona, Spain) into each nostril; four swabs per animal were taken and placed into a tube with 4 mLof 1× PBS (VWR; Barcelona, Spain). Then, the tubes were shaken and incubated overnight at 4 °C. The swabs were discarded and the tubes were centrifuged at 2000 rpm to obtain the supernatant, which was stored at −20 °C until use. For saliva samples, four swabs per animal were also used to collect the sample; these samples were processed following the same protocol as the nasal secretions [16]. 

### 2.3. Antigen Production

#### 2.3.1. Somatic Antigen from Third-Stage Larvae of *T. circumcincta*

Somatic antigen of *T. circumcincta* L_3_ (L_3_SE-Tc) was prepared based on Sinski et al. [23], with slight modifications. Briefly, 500,000 *T. circumcincta* L_3_ were washed in cold sterile PBS and then mashed in liquid nitrogen with a hand-held homogenizer. The mixture was diluted in an inhibition solution (1mM EDTA, 1 mM PMSF, and 0.05 M of Tris-HCl) and homogenized using an ultrasound (80%, 0.5 s, 10 cycles). The mixture was frozen for 60 min at −80 °C and centrifuged at 8000 rpm for 60 min. The supernatant was sterilized via filtration through a 0.22 µm pore diameter filter (Sigma-Aldrich; Madrid, Spain). The protein concentration was estimated using the Bradford method with bovine serum albumin (BSA) standards and then stored in 50 µL aliquots [42]. 

#### 2.3.2. Recombinant Protein Disulfide Isomerase of *T. circumcincta*

The 203-amino-acid sequence of the protein disulfide isomerase of *T. circumcincta* (PDI-Tc) was previously described by Martínez-Valladares et al. [35] (DQ357222.1). The recombinant protein of this fragment was produced by Proteogenix (Strasbourg, France). In brief, the cDNA sequence was cloned in expression vector pQE30 and expressed in *Escherichia coli*. Protein purification was performed by affinity chromatography against His-tag on nickel resin. Elution was carried out using imidazole buffer. The concentration of extracted protein was measured viathe Bradford method.

### 2.4. Indirect ELISA against Gastrointestinal Nematodes

An indirect ELISA, using either L_3_SE-Tc or PDI-Tc antigen, was performed according to Martínez-Valladares et al. [38] and Atlija et al. [41], with slight modifications. Microtiter plates (BRAND plates^®^immunoGrade; Madrid, Spain) were coated with 100µLof PBS containing 2.5 µg/mL of L_3_SE-Tc or 5 µg/mL of PDI-Tc and were incubated overnight at 4 °C. After discarding the contents, the plates were blocked using 200 µL of 4% PT-milk (4 g of powdered milk + 100 mL PBS–Tween20) (PBS–Tween20: 1 L PBS; pH = 7.4 + 1 mL Tween20) and incubated at 37 °C for 30 min. Then, the blocking buffer was discarded and the plates were washed four times for the L_3_SE-Tc assays or six times for the PDI-Tc assays using 200 µL of PBS-Tween20. A total of 100 µL of each sample was prepared and added following each dilution, as shown in Table 2. The plates were incubated with each sample at 37 °C for 30 min or 45 min in the case of L_3_SE-Tc or PDI-Tc, respectively. Then, the plates were washed as previously described, and 100 µL of Rabbit Anti-Sheep IgA-HRP (Abcam;Cambridge, United Kingdom;) was added and diluted in blocking buffer; the used dilution was 1/500 for all samples and antigens, with the exception of the serum against PDI-Tc, which was 1/250. The plates were incubated at 37 °C for 30 min for L_3_SE-Tc and 45 min for PDI-Tc. After the final wash step, 100 µL of 3,3′,5,5′-Tetramethylbenzidine (Sigma-Aldrich; Madrid Spain) substrate was added and incubated at 25 °C for 10 min. Then, the reaction was stopped by the addition of 100 µL of 2M H_2_SO_4_. Absorbance was measured at 450 nm. To ensure the standardization of IgA measurement, positive and negative controls were included in each plate. 

The optical density (OD) index was calculated as follows:

$$\mathrm{OD}\;\mathrm{index}\;\frac{\mathrm{OD}\;\mathrm{sample}\;-\;\mathrm{OD}\;\mathrm{negative}}{\mathrm{OD}\;\mathrm{positive}-\;\mathrm{OD}\;\mathrm{negative}}$$

### 2.5. Statistical Analysis

Extreme values were removed (Mean ± 3SD) to minimize the inclusion of extreme values. A Kolmogorov–Smirnov test was employed to determine if data were adjusted to a normal distribution. All ewes were classified according to their individual FEC in four different phenotypes (F1: 0–14 epg; F2: 15–100 epg; F3: 115–300 epg; F4: >300 epg) and then the proportion of animals for each phenotype and breed was calculated. A Kruskal–Wallis test was used to determine if there were significant differences between these four phenotypes. Associations between FEC and OD were measured using Spearman’s correlation rank. The significance level was set at *p* < 0.05.

Due to the high percentage of ewes with 0 epg, the risk of being infected depending on different variables (age, breed, type of pasture) was measured, and a multilevel model to fit the logit of the odds (log probability of being infected/probability of not being infected) was constructed and tested using the GLIMMIX procedure of SAS (SAS Institute Inc., Cary, NC, USA). The model included the breed (Assaf, Churra, and Castellana), the age of the animals (Young < 12 months; Adult > 12 months), and the interactions as fixed effects. Pearson chi-squared/DF values were nearest to 1 (0.99–1.01), suggesting no effect of overdispersion on probability values.

## 3. Results

### 3.1. Fecal Egg Count and Risk of Infections Depending on the Breed

**Figure 1:**
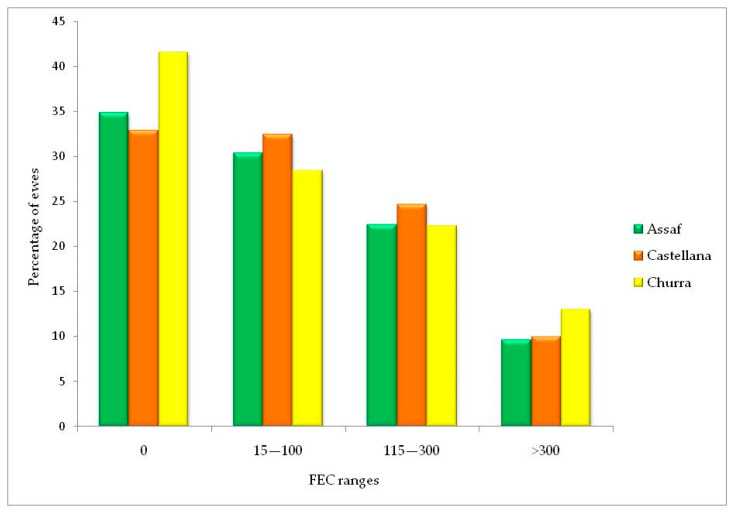
Proportion of Assaf, Castellana, and Churra animals classified according to their individual fecal egg counts (FEC) in four different phenotypes.

After measuring the individual level of infections in all sampled ewes (*n* = 589), the extreme data were removed and the final sample size was 193 young ewes and 394 adult ewes (*n* = 582 in total). FEC values ranged from 0 to 975 epg depending on the age and sheep breed. The percentage of ewes with a value of 0 for the FEC represented 64.66% of ewes sampled (20.58% of young ewes and 44.08% of adult ewes) (Table 3). The percentage of ewes belonging to each phenotype according to their FEC is shown in Figure 1. After morphological identification of L_3_, most of them were classified as *T. circumcincta* (21–100%) and *Trichostrongylus* ssp. (15–85%). For percentages lower than 10%, other species were identified as *Chabertia ovina* or *Oesophagostomum* spp.

### 3.2. Prediction of Infection Risk

A multilevel random intercept model was used to predict the influence of breed and age factors on predicting risk of infection (FEC > 0). According to this model, breed is not an influential factor in this study. However, age was considered a significant factor as the probability of infection was 2.39 times higher in young animals, although not in the same way in all breeds; while the Churra and Assaf breeds showed this differentiation between young and adult ewes, age did not affect the probability of infection in the Castellana breed (Table 4). 

The type of pasture (communal or owned pastures) was included as a possible influential factor in the probability of infection. This model showed that animals grazing in communal pastures had a higher probability of infection than those who did not share pasture land (Table 4).

### 3.3. IgA Levels against GIN as a Marker of Infection Level

Since the multilevel random intercept model showed the variable of breed did not affect the probability of infections, breed was not taken into account in the analysis of IgA and its possible association with FEC (Table 5). IgA detected in serum samples showed a significant positive correlation with FEC using the L_3_SE-Tc antigen in both groups, with a stronger association in the young ewes (Rho = 0.306; *p* = 0.00) than in the adult group (Rho = 0.123; *p* = 0.00); using the PDI-Tc antigen, no significant correlations were detected in this sample. 

The association between FEC and IgA in the nasal secretions of young ewes was positive, high, and similar for both the L_3_SE-Tc (Rho = 0.504; *p* = 0.00) and PDI-Tc (Rho = 0.498; *p* = 0.00) antigens. In adult ewes, a significant correlation between these variables was only present using the PDI-Tc antigen (Rho = 0.179; *p* = 0.00). 

Regarding the saliva samples, a unique association was found between FEC and IgA levels in the young group of ewes (Rho = 0.295; *p* = 0.00) when using the PDI-Tc antigen.

### 3.4. Association between IgA Levels for the Different Biological Samples

Associations between IgA levels measured by the two indirect ELISAs against the L_3_SE-Tc or PDI-Tc antigens were evaluated for each type of biological sample. Correlations between these ELISAs were found in nasal secretions (Rho= 0.497; *p* = 0.00) as well as in saliva (Rho = 0.149; *p* = 0.00). A correlation was not found in the serum samples. Interestingly, in the nasal secretions, this association between ELISAs was kept in both groups, in young (Rho = 0.554; *p* = 0.00) and adult ewes (Rho = 0.441; *p* = 0.00).

In addition, the association between the IgA levels in the different types of samples was measured. The serum showed a positive correlation using the L_3_SE-Tc antigen only with saliva (Rho = 0.183; *p* = 0.00). However, using PDI-Tc antigen, the serum showed positive correlations with saliva (Rho = 0.146; *p* = 0.00) and also with nasal secretions (Rho = 0.110; *p* = 0.00). A correlation was found between saliva and nasal secretions using both L_3_SE-Tc (Rho = 0.178; *p* = 0.00) and PDI-Tc (Rho = 0.255; *p*= 0.00) (Table 6A,B) antigens.

## 4. Discussion

The development of more sustainable control methods has become an urgent need due to the increase of anthelmintic resistance in GINs infecting ruminants. Under this premise, it is essential to understand the mechanism through which some ruminants can control GIN infections in a better way than others. The immune response against GIN involves the production of IgA, and its increase has been related to more resistant sheep under experimental conditions, especially against *T. circumcincta* and *H. contortus*. Indeed, in experimentally infected sheep, increasedlevels of IgA in adult Churra sheep and lambs belonging to the Scottish Blackface and Canarian Hair breeds have been associated with shorter female nematodes and, consequently, with a lower number of female worm eggs in utero [25,43,44]. Most of the IgA present in saliva is produced by B-lymphocytes that migrate through the blood from the lymphoid tissue associated with gastrointestinal mucosa; however, the relationship between IgA in saliva and plasma remains to be determined [45,46]. Saliva and nasal secretions are biological samples more easily accessible than blood and less invasive for animals. Moreover, analysis of antibody levels can be performed in the laboratory on a large scale in a very short time, overcoming the difficulties of conventional techniques, such as coprological methods [27,47,48]. 

The development of immunity to GIN in sheep is complex and highly variable among breeds, and also among individuals belonging to the same breed. Consequently, breed has been suggested as an important factor in immune control, despite most breeds having been poorly studied. According to some studies performed under experimental conditions, certain breeds are considered more resistant than others to GIN infections; in particular, hair sheep breeds (Canarian Hair breed, Blackbelly, or Pelibuey) in comparison to wool breeds (Suffolk, Canarian Sheep) [49,50,51,52,53]. Based on the Spanish Ministry of Agriculture, Fisheries and Food database, the most common sheep breeds in the sampling area of the present study are Assaf and the autochthonous breeds Castellana and Churra, all of them being wool breeds. In the literature, there is no information about how these three breeds are able to control GIN infections under natural conditions. In this study, the results showed that the infection risk is not linked to any of these breeds. 

On the other hand, age is another variable to be considered. The development of immunity is variable. Lambs start to show immune competence from 2–3 months of age, reaching apeak at 12 months when protective immune capacity is fully developed [54,55]. As expected, in this study, infection risk was associated with age, being higher in young ewes, but not in the same way for the three breeds, since there was no difference regarding the risk of infection between young and adult Castellana ewes.

In experimental infections, negative correlations have been described between FEC and IgA levels in serum samples measured using the L_4_ somatic antigen of *T. circumcincta* or PDI-Tc in Churra sheep, but also in Blackface and Soay sheep using the L_3_ somatic antigen of *T. circumcincta* [16,56,57,58,59]. However, the relationship between FEC and IgA in natural infections is not clear yet; Shaw et al. [60] measured specific IgA in saliva against the CarLA antigen and detected a negative association with FEC in Romney and Texel cross lambs. CarLA is a surface antigen from *Trichostrongylus colubriformis* and presents only in the L_3_ stage. A commercial test, called the CARLA TEST, is used to select those animals that are able to control infection in a better way than others. However, De la Chevrotére [61] used the excretory/secretory antigens of L_3_ *H. contortus* and showed that IgA levels measured in the serum samples of goat kids naturally infected with *H. contortus* correlated positively with FEC. In the current study, all the associations between FEC and IgA were positive, being stronger in young ewes for all samples. In the serum and saliva samples, these associations were moderate against L_3_SE-Tc (Rho = 0.306; *p* = 0.000) and PDI-Tc (Rho = 0.295; *p* = 0.000); however, they were stronger in nasal secretions, regardless of the antigen used, either the L_3_SE-Tc antigen (Rho = 0.504; *p* = 0.00) or the PDI-Tc antigen (Rho = 0.498.; *p* = 0.000). These data suggest that young ewes with a higher FEC produce more IgA under natural conditions, where the animals are continuously exposed to GIN infections. Accordingly, IgA from nasal secretions could be a possible GIN infection marker in young ewes. 

The sheep that are infected under natural conditions are usually infected by mixed-nematode species [62]. Among these species, some, such as *H. contortus* or *T. circumcincta*, are more pathogenic or prevalent than others, and therefore their control is of greater interest [63,64]. Generally, IgA detection in sheep and goats using somatic and excretory/secretory antigens is not highly specific and presents an extensive cross-reactivity between species of the Trichostrongylidae family, such as *H. contortus*, *T. circumcincta,* and *Trichostrongylus* spp. [65,66]. Cuquerella et al. [67] found an intense cross-reaction in the serum of lambs infected with *T. circumcincta* using somatic extract of *H. contortus* adults. Smith et al. [68] isolated a membrane glycoprotein of *T. circumcincta* that was also recognized by *H. contortus*. Additionally, Martinez-Valladares et al. [35] were the first to produce a recombinant fragment of the PDI protein in *T. circumcincta* and to describe a cross-reaction with *Trichostrongylus* spp. This has conditioned the use of the ELISA test as a specific diagnostic method. Some studies have tried to solve cross-reactions between some species, such as *T. colubriformis*, *H. contortus*, *T. circumcincta*, *Cooperia curticei*, and *Nematodirus spathiger*, using recombinant antigens, but not always successfully [69]. However, the low specificity of these antigens could be beneficial in natural conditions, where the aim is to detect the infection caused by any of these GIN species. In this study, the most frequent species were *T. circumcincta* and *Trichostrongylus* spp. in all flocks; therefore, any of the antigens tested in this study might be able to detect antibodies against both species. Consequently, the application of these tests would depend on the GIN population infecting sheep. Further studies are needed to confirm their utility with another mix of species.

IgA is the most common antibody in saliva fluid [46]. It is produced by plasma cells that are supposed to originate from lymphoid tissue in the intestinal mucosa and then migrate via the circulatory system to salivary-duct-associated lymphoid tissue [20,46]. This seems to be the explanation as to why IgA levels against GIN in saliva reflect the immune response in gastric mucosa in infected sheep [20]. We measured the correlation between IgA levels in serum and saliva to confirm if there is a direct relationship. Our results showed a slight positive correlation with both antigens (L_3_SE-Tc; Rho = 0.183; *p* = 0.00 and PDI-Tc; Rho = 0.146; *p* = 0.00). Escribano et al. [33] detected an increase in IgA levels measured in the serum and saliva ofsheep experimentally infected with *H. contortus.* These authors hypothesized that IgA in saliva could recognize and bind L_3_ during the intake and then act together with mast cells or eosinophils once they reach the gut, limiting larval number, maturation, and egg shedding. 

## 5. Conclusions

Specific IgA against GIN was positively associated with FEC in naturally infected sheep, with the nasal secretions from young animals providing the samples where this association was stronger, which could therefore be used as markers of infection. Moreover, the predictive model explained that variation in the risk of infection is not influenced by any of the breeds included in this study—Assaf, Castellana, and Churra. However, as expected, the model showed that age was a key factor and young animals have a greaterrisk of infection than adult ewes, except for the Castellana breed, where all animals showed the same risk regardless of age.

## Figures and Tables

**Table 1:** Number of ewes (*N*), breed, and type of pasture for each flock. Communal land is shown as CL, while owned land is shown as OL.

Flock	Breed	*N*	Type of Pasture
**1**	Churra	42	CL
**2**	Churra	40	CL
**3**	Assaf	13	OL
**4**	Assaf	40	CL
**5**	Assaf	24	OL
**6**	Assaf	46	CL
**7**	Churra	48	CL
**8**	Castellana	40	CL
**9**	Castellana	40	CL
**10**	Assaf	40	CL
**11**	Castellana	53	CL
**12**	Assaf	36	OL
**13**	Castellana	40	CL
**14**	Castellana	41	CL
**15**	Castellana	43	OL
**Total**		589	

**Table 2:** Dilutions used for each type of sample and antigen.

	L_3_SE-Tc		PDI-Tc	
	Sample	Antibody	Sample	Antibody
**ELISA-Saliva**	1/3	1/500	1/2	1/500
**ELISA-Nasal secretions**	1/4	1/500	1/2	1/500
**ELISA-Serum**	1/1	1/500	1/2	1/250

**Table 3:** Size, mean, range, and percentage of positives for each breed, age, and total of ewes.

			FEC	
Breed		Total Ewes	Young Ewes	Adult Ewes
**Assaf**	**Mean ± SD** **Range**	132 ± 180 (0–975)	122 ± 159 0–825	128 ± 181 0–975
	**% positive FEC**	64.97%	62.22%	65.79%
	***N***	197	45	152
**Castellana**	**Mean ± SD** **Range**	108 ± 162 0–915	99 ± 140 0–735	113 ± 164 0–915
	**% positive FEC**	67.06%	58.76%	75.19%
	***N***	255	126	129
**Churra**	**Mean ± SD** **Range**	151 ± 181 0–945	155 ± 157 0–735	151 ± 181 0–945
	**% positive FEC**	59.68%	81.81%	54.63%
	***N***	129	22	108
**Total ewes**	**Mean ± SD**	113 ± 173	108 ± 164	113 ± 173
	**Range**	0–975	0–825	0–975
	**% positive FEC**	64.43%	85.50%	54.24%
	***N***	582	193	389

**Table 4:** Odds ratios of factors explaining the prevalence ofinfection by *T. circumcincta*. ^1^ Lead mean square on the probability scale (prevalence). ^2^ Odds ratio using the Churra breed as a reference level. Significance is shown as * *p* < 0.05 and ** *p* < 0.01.

	Odds Ratio (95% CI)	*p*-Value	Estimated Prevalence ^1^
**Breed**			
Assaf	**Reference**		0.5976
Castellana	0.76 (0.46–1.24)	0.2684	0.5285
Churra	0.69 (0.33–1.44)	0.3221	0.5053
Castellana	1.10 (0.56–2.13) ^2^	0.7835	
**Age**			
Adult ewes	**Reference**		0.4354
Young ewes	2.39 (1.37–4.19)	0.0022 *	0.6488
**Breed × Age**			
AdultAssaf	**Reference**		0.4550
Young Assaf	3.16 (1.35–7.43)	0.0083 *	0.7255
Adult Castellana	**Reference**		0.5391
Young Castellana	0.92 (0.51–1.67)	0.7806	0.5180
Adult Churra	**Reference**		0.3197
Young Churra	4.72 (1.42–15.7)	0.0115 *	0.6895
**Pasture type**			
Farm pastures	**Reference**		0.3113
Communal pastures	6.97 (3.84–12.6)	0.0001 **	0.7592

**Table 5:** Spearman’s rank correlation between fecal egg count (FEC) and IgA levels for each antigen. *N*: number of ewes.

	Young Ewes (*N* = 193)		Adult Ewes (*N* = 389)	
	L_3_SE-Tc	PDI-Tc	L_3_SE-Tc	PDI-Tc
**Serum**	Rho = 0.306 ** p = 0.000	Rho = 0.107 *p* = 0.159	Rho = 0.123 ** p = 0.000	Rho = 0.091 *p* = 0.077
**Nasal**	Rho = 0.504 ** p = 0.000	Rho = 0.498 ** p = 0.000	Rho = 0.031 *p* = 0.554	Rho = 0.179 ** p = 0.000
**Saliva**	Rho = 0.058 *p* = 0.137	Rho = 0.295 ** p = 0.000	Rho = 0.059 *p* = 0.246	Rho = 0.001 *p* = 0.989

** Significant correlations (*p* < 0.000).

**Table 6:** (**A**) Spearman’s rank correlations between IgA levels measured for each sample using the L_3_SE-Tc antigen. (**B**) Spearman’s rank correlations between IgA levels measured foreach sample using the PDI-Tc antigen.

**(A)**			**L_3_SE-Tc**	
		Serum	Nasal secretions	Saliva
**L_3_SE-Tc**	Serum	1		
	Nasal secretions	Rho = 0.05 *p* = 0.205	1	
	Saliva	Rho = 0.183 ** p = 0.000	Rho = 0.178 ** p = 0.000	1
**(B)**			**PDI-Tc**	
		Serum	Nasal secretions	Saliva
**PDI-Tc**	Serum	1		
	Nasal secretions	Rho = 0.110 ** p = 0.000	1	
	Saliva	Rho = 0.146 ** p = 0.000	Rho = 0.255 ** p = 0.000	1

** Significant correlations (*p* < 0.00).

## Data Availability

The data supporting the conclusions of this article will be made available by the authors, under reasonable request.
